# Women with polycystic ovary syndrome are burdened with multimorbidity and medication use independent of body mass index at late fertile age: A population‐based cohort study

**DOI:** 10.1111/aogs.14382

**Published:** 2022-06-08

**Authors:** Linda Kujanpää, Riikka K. Arffman, Paula Pesonen, Elisa Korhonen, Salla Karjula, Marjo‐Riitta Järvelin, Stephen Franks, Juha S. Tapanainen, Laure Morin‐Papunen, Terhi T. Piltonen

**Affiliations:** ^1^ PEDEGO Research Unit (Research Unit for Pediatrics, Dermatology, Clinical Genetics, Obstetrics and Gynecology) University of Oulu Oulu Finland; ^2^ Medical Research Center Oulu (MRC Oulu) University of Oulu Oulu Finland; ^3^ Department of Obstetrics and Gynecology Oulu University Hospital Oulu Finland; ^4^ Infrastructure for Population Studies, Faculty of Medicine University of Oulu Oulu Finland; ^5^ Department of Epidemiology and Biostatistics, MRC‐PHE Center for Environment and Health School of Public Health, Imperial College London London UK; ^6^ Center for Life Course Health Research, Faculty of Medicine University of Oulu Oulu Finland; ^7^ Unit of Primary Health Care Oulu University Hospital Oulu Finland; ^8^ Department of Life Sciences, College of Health and Life Sciences Brunel University London London UK; ^9^ Institute of Reproductive and Developmental Biology Imperial College London London UK; ^10^ Department of Obstetrics and Gynecology University of Helsinki, Helsinki University Hospital Helsinki Finland

**Keywords:** cohort study, comorbidity, medication use, multimorbidity, polycystic ovary syndrome

## Abstract

**Introduction:**

This population‐based follow‐up study investigated the comorbidities, medication use, and healthcare services among women with polycystic ovary syndrome (PCOS) at age 46 years.

**Material and methods:**

The study population derived from the Northern Finland Birth Cohort 1966 and consisted of women reporting oligo/amenorrhea and hirsutism at age 31 years and/or a PCOS diagnosis by age 46 years (*n* = 246) and controls without PCOS symptoms or diagnosis (*n* = 1573), referred to as non‐PCOS women. The main outcome measures were self‐reported data on symptoms, diagnosed diseases, and medication and healthcare service use at the age of 46 years.

**Results:**

Overall morbidity risk was increased by 35% (risk ratio [RR] 1.35, 95% confidence interval [CI] 1.16–1.57) and medication use by 27% [RR 1.27, 95% CI 1.08–1.50) compared with non‐PCOS women, and the risk remained after adjusting for body mass index. Diagnoses with increased prevalence in women with PCOS were migraine, hypertension, tendinitis, osteoarthritis, fractures, and endometriosis. PCOS was also associated with autoimmune diseases and recurrent upper respiratory tract infections and symptoms. Interestingly, healthcare service use did not differ between the study groups after adjusting for body mass index.

**Conclusions:**

Women with PCOS are burdened with multimorbidity and higher medication use, independent of body mass index.

**Abbreviations**
BMI: body mass index
HA: hirsutism
MET: metabolic equivalent of task
NFBC1966: Northern Finland Birth Cohort 1966
NW: normal weight
OW/OB: overweight/obese
PCOM: polycystic ovary morphology
PCOS: polycystic ovary syndrome

**Key message**

Women with polycystic ovary syndrome are burdened with multimorbidity and higher medication use, independent of body mass index.

## 1 INTRODUCTION

Polycystic ovary syndrome (PCOS) is the most common endocrine disorder in women of reproductive age, with a prevalence of 5%–18%.^1^, ^2^, ^3^ PCOS used to be mainly considered a reproductive condition; however, it is now commonly accepted that women with PCOS are at high risk for metabolic disorders, such as obesity,^4^, ^5^ impaired glucose tolerance, type 2 diabetes mellitus (DMT2),^6^ metabolic syndrome,^7^ and, possibly, cardiovascular events.^8^, ^9^ Furthermore, women with PCOS have been shown to have an increased risk for psychological morbidity,^10^, ^11^ asthma,^12^ and migraine.^13^

Surprisingly, only a few studies have systematically assessed the overall comorbidity in women with PCOS.^13^, ^14^ This should be of high priority given the high cost to society resulting from PCOS‐related morbidity. For example, PCOS‐related DMT2 alone has been estimated to carry an annual cost of £237 million ($310 million) in the UK^15^ and $1.77 billion in the USA.^16^ Despite PCOS being a common condition and carrying a high morbidity risk, the syndrome often remains underdiagnosed and is therefore underrepresented in patient records and national registers, which limits capturing comorbidities.^1^, ^17^ Previous studies have primarily reported hospital‐based diagnoses with no data on symptoms. Moreover, the focus has been mainly on women in their early or mid‐reproductive years, and morbidity data on late fertile age are scarce. The present study focused on assessing morbidities, self‐reported symptoms, medication use, and the use of healthcare services in 46‐year‐old women with PCOS and in non‐PCOS controls who were part of the population‐based Northern Finland Birth Cohort (NFBC).

## 2 MATERIAL AND METHODS

### 2.1 Study population

The study population was derived from the longitudinal Northern Finland Birth Cohort 1966 (NFBC1966),^18^, ^19^ including all pregnancies with estimated date of delivery during 1966 in the two northernmost provinces of Finland (5889 females). There have been several pre‐set data collection points determined by the cohort center. The data collection and identification of PCOS cases have been previously described.^10^ In brief, at age 31 years, questions were asked about PCOS‐related symptoms, oligo/amenorrhea, and hirsutism and at age 46 years the women were asked if they had ever been diagnosed with polycystic ovaries (ie, polycystic ovary morphology; PCOM) and/or PCOS. The whole PCOS population (*n* = 280, 4.8% of the population) consisted of women who reported both hirsutism and oligo/amenorrhea at age 31 (4.1%) and/or PCOM/PCOS at age 46 (3.1%), of which 246 replied to the 46‐year questionnaire. The non‐PCOS control population consisted of all the remaining women (no PCOS symptoms at age 31 and no self‐reported PCOS diagnosis by age 46, *n* = 1573). Women who were pregnant or using hormonal contraceptives at age 31 (*n* = 1488) were excluded because of possible bias towards oligo/amenorrhea. Moreover, women who did not permit the use of their data were excluded (n_31_ = 41 and n_46_ = 14) (Supporting Information Figure S1). The validity of identifying women with PCOS in the NFBC1966 data has been verified in previous publications.^5^, ^10^ The study flow chart is presented in Figure S1.

### 2.2 Questionnaires on morbidity and symptoms

A questionnaire was sent to participants at the age of 46. Morbidity was assessed via the following question: “Do you currently have, or have you ever had, any of the following symptoms, diseases, or injuries diagnosed or treated by a doctor?” (Table 1). The diagnoses were chosen by the cohort center and the involved investigators and were organized according to the World Health Organization International Classification of Diseases 10th revision coding system (Table 1). For osteoarthrosis diagnoses, all locations (hip, back, knee, finger, joint, foot, shoulder, other) were combined for “Any arthrosis” analysis.

**TABLE 1:** Self‐reported diagnosis at age 46 years

Self‐reported diagnosis at age 46	Control, *n* (%)	PCOS, *n* (%)	cOR (95% CI)	aOR (95% CI)
Diabetes type 2	34 (2.2%)	16 (6.7%)	3.21 (1.74–5.91)*	**2.40 (1.25–4.63)**
Depression	219 (14.0%)	49 (20.5%)	1.58 (1.12–2.23)*	**1.57 (1.09–2.25)**
Migraine	386 (24.7%)	82 (34.2%)	1.59 (1.19–2.12)*	1.58 (1.17–2.13)*
Hypertension	230 (17.9%)	73 (30.4%)	2.00 (1.48–2.71)*	1.76 (1.27–2.44)*
Tendinitis	150 (9.6%)	40 (16.9%)	1.91 (1.30–2.95)*	1.81 (1.22–2.68)*
Any arthrosis	280 (18.1%)	64 (26.8%)	1.66 (1.21–2.27)*	1.66 (1.20–2.29)*
Fractures	263 (16.8%)	58 (24.5%)	1.61 (1.16–2.22)*	1.73 (1.24–2.41)*
Endometriosis	131 (8.4%)	32 (13.4%)	**1.70 (1.12–2.56)**	1.81 (1.19–2.76)*
Gestational diabetes	344 (25.1%)	65 (30.2%)	2.00 (1.48–2.71)*	1.52 (0.97–2.37)
Pre‐eclampsia	117 (8.7%)	33 (15.5%)	1.94 (1.28–2.94)*	**1.76 (1.15–2.71)**

*Note*: The results are reported as crude odds ratios (cORs) with 95% confidence intervals (CIs) and adjusted odds ratios (aORs) with 95% confidence intervals (CIs). The ORs in bold and the ones marked with * (Benjamini–Hochberg correction) were statistically significant. Confounding factors used for adjustments: Body mass index, physical activity, alcohol consumption, smoking, marital status, and education.

Regarding respiratory/allergic symptoms, the questionnaire included the question: “Have you had the following respiratory and/or allergic symptoms or diseases?” The answer options were Never; Yes, within the last 12 months; and Yes, but more than a year ago. Affirmative answers were combined to form a new variable, which was used for the analysis. The questionnaire also included two questions about symptoms of infection and autoimmune diseases, with no diagnostic data. The items are listed in Table 2.

**TABLE 2:** Self‐reported symptoms concerning allergies, infections, and autoimmune symptoms at age 46 years

	46 years			
	Controls, *n* (%)	PCOS, *n* (%)	cOR (95% CI)	aOR (95% CI)
Questions concerning asthma and allergy				
Cough with wheezing	450 (29.5%)	98 (41.9%)	1.72 (1.30–2.28)*	1.70 (1.27–2.28)*
Recurrent respiratory infections	429 (28.6%)	85 (37.9%)	1.53 (1.14–2.05)*	1.54 (1.15–2.08)*
Emphysema, chronic bronchitis	68 (4.5%)	16 (6.9%)	1.58 (0.90–2.77)	1.62 (0.92–2.87)
Atopic, infantile, or allergic eczema	433 (28.3%)	84 (36.4%)	1.45 (1.08–1.93)*	1.45 (1.08–1.95)*
Questions concerning infection symptoms				
Pneumonia at least twice	137 (8.8%)	37 (15.4%)	1.88 (1.27–2.78)*	1.91 (1.28–2.86)*
Recurrent otitis in adulthood	67 (4.3%)	25 (10.4%)	2.55 (1.58–4.12)*	2.53 (1.53–4.17)*
Other health‐endangering recurrent infections	31 (2.0%)	11 (4.6%)	2.29 (1.14–4.61)*	2.27 (1.11–4.63)*
More common colds	97 (6.2%)	25 (10.4%)	1.75 (1.10–2.78)*	1.67 (1.28–3.54)*
More susceptible to infections than other people	74 (4.7%)	22 (9.2%)	2.07 (1.26–3.40)*	2.13 (1.28–3.54)*
Questions concerning autoimmune symptoms				
Joint pain	572 (36.4%)	113 (47.3%)	1.57 (1.19–2.06)*	1.46 (1.10–1.94)*
Joint swelling	240 (15.3%)	53 (22.2%)	1.57 (1.13–2.20)*	1.49 (1.05–2.11)*
Pain of the heel	278 (17.8%)	71 (29.6%)	1.94 (1.43–2.64)*	1.75 (1.26–2.41)*

*Note*: The results are reported as crude odds ratios (cORs) and adjusted (body mass index, physical activity, alcohol consumption, smoking, marital status, and education) odds ratios (aORs) with 95% confidence intervals (CIs). The OR values in bold and the ones marked with * (Benjamini–Hochberg correction) were statistically significant.

### 2.3 Overall morbidity score and self‐reported health status

To create an overall morbidity score, a sum score of all the Yes answers from the questions regarding morbidities diagnosed by a doctor (*n* = 68) was calculated. Perceived health status at age 46 years was assessed by the following question: “How would you estimate your current state of health?”. Answer options were very good, good, moderate, poor, and very poor. Answer options 1 and 2 and options 4 and 5 were merged for the analysis.^20^

### 2.4 Use of health services

The following question concerning the use of public health services at the age of 46 was used: “How many times have you consulted the following healthcare providers or professionals because of your illness or symptoms during the past year?”. The options covered visits/appointments to a doctor, public health nurse, psychologist, physiotherapist, dentist, or other specialist working in a public health center, occupational health service, special welfare institution, or other institution, such as a private clinic. The sum scores of all visits and visits to any doctor were analyzed separately.

### 2.5 Medication and supplement use

**FIGURE 1:**
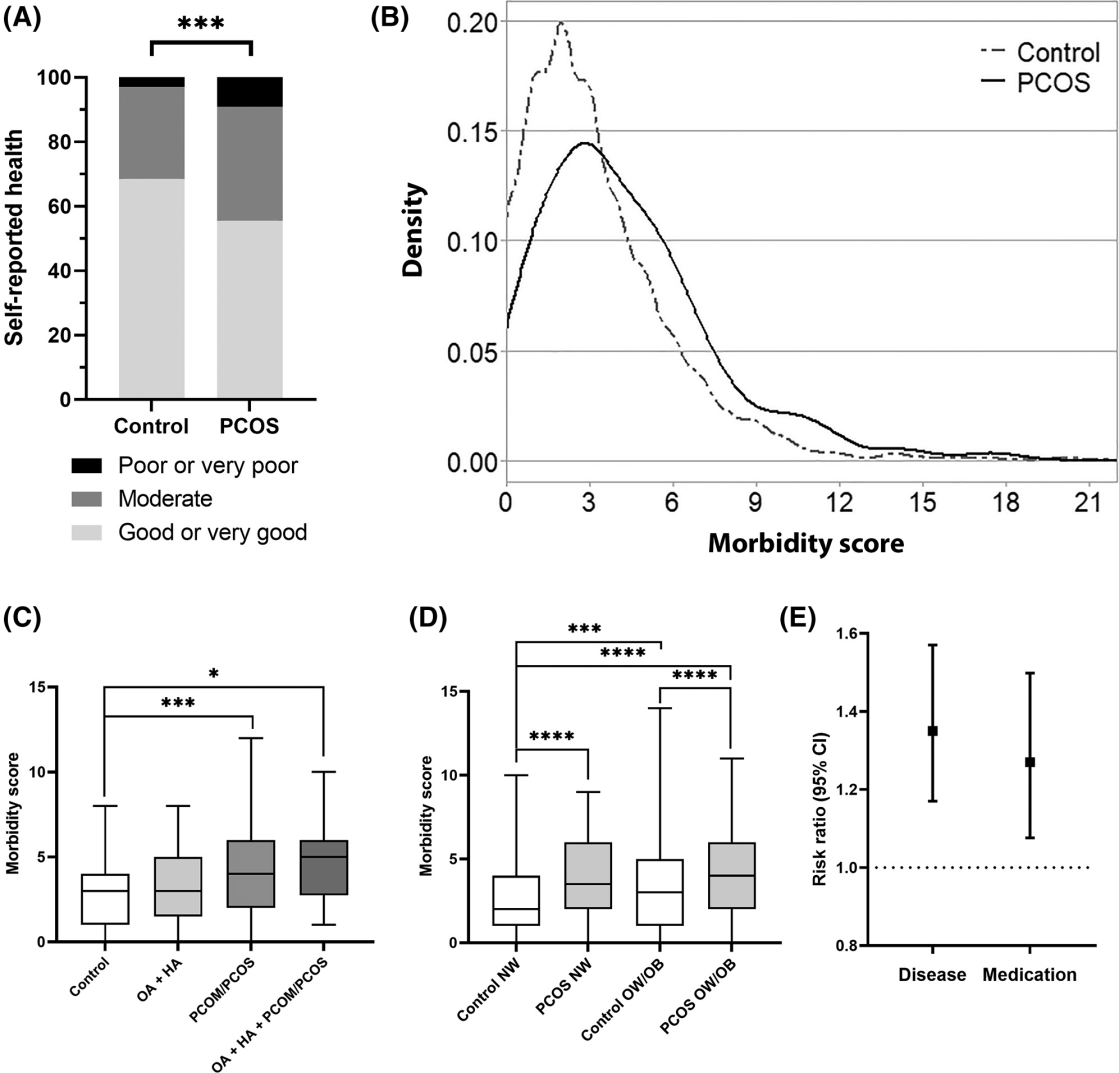
Morbidity related to PCOS. (A) The proportion (%) of control women and women with PCOS reporting their current health status at age 46 years. (B) Distribution of morbidity scores at age 46 in women with PCOS (black) and non‐PCOS controls (dark gray). (C) Comparison of morbidity at age 46 between different PCOS phenotypes (OA + HA reported at 31, PCOM/PCOS reported at 46 or OA + HA at 31 + PCOM/PCOS at 46). (D) The comparison of morbidity scores between different weight groups PCOS vs control. NW = BMI <25 kg/m^2^, OW/OB = BMI ≥25 kg/m^2^ (E) The risk ratio with 95% confidence interval (CI) for any diagnosis or medication at age 46 in women with PCOS in comparison to the non‐PCOS controls. In (C) and (D) the boxes represent interquartile ranges and the line represents the median. The whiskers show the minimum–maximum values, after exclusion of outliers. Abbreviations: BMI, body mass index; HA, hirsutism; NW, normal‐weight; OA, oligo/amenorrhea; OW/OB, overweight/obese; PCOM, polycystic ovary morphology; PCOS, polycystic ovary syndrome. *p < 0.05, ***p < 0.005, ****p < 0.0001

Medication use was determined with an open‐ended question, and the data were classified according to the international Anatomical Therapeutic Chemical (ATC) classification. The data were analyzed as the main ATC groups, and if the main group was significantly different between the PCOS cases and the controls, the medication subgroups were also analyzed. The risk for medication use was calculated based on the sum score of the medication main groups used. The medication data are reported in Figure 1D and Supporting Information Table S1.

### 2.6 Confounding factors

All the confounding variables included in the study represent factors commonly considered to relate to exposure or overall strongly to morbidity (Supporting Information Table S1). Body mass index (BMI) was categorized into three groups: <25 kg/m^2^, 25–30 kg/m^2^, and >30 kg/m^2^.^5^ Physical activity was calculated as the metabolic equivalent of task (MET) scores in hours per week from the frequency and duration of leisure time activities (3 METs = light physical activity and 5 METs = brisk physical activity).^21^ Alcohol consumption was categorized into three groups: abstinence, low‐risk drinking (≤20 g/day) or high‐risk drinking (>20 g/day).^22^ Smoking was categorized as no smoking, previous/occasional smoking, or regular smoking. Marital status was categorized into two groups: single or in a relationship. Socioeconomic status was classified into three categories based on education level: basic, secondary, and tertiary.

### 2.7 Missing data and statistical analyses

Data were considered missing if the participant had not answered a question at all, ie had not chosen any of the answer options. Data were missing for 0.6%–3.3% of the women. For the self‐reported symptoms, 3.4%–5.2% of the participants did not answer the question. As the percentages were low, the missing data were not imputed.

Statistical analyses were conducted using IBM‐SPSS Statistics (version 24) for Windows software, and figures were produced using graphpad prism (version 7.03) and R (version 4.0.3) software. A value of *p* less than 0.05 was considered statistically significant. Differences in the categorical variables between the study groups were analyzed using Pearson's chi‐squared test. The specific group differences were deciphered by adjusted standardized residual counts. The continuous variables were tested for normal distribution using the Kolmogorov–Smirnov test. The independent samples *t* test or the Mann–Whitney *U* test were used to analyze the differences in continuous variables. The associations between self‐reported diagnoses as well as symptoms and medication use among the PCOS group vs the control group were analyzed with binary logistic regression models. The adjusted logistic regression models were performed by including the following variables in the models: BMI, physical activity, alcohol consumption, smoking, marital status, and education. To control for type I errors due to multiple‐hypothesis testing, values of *P* below the false discovery rate calculated by the Benjamini–Hochberg method were considered as statistically significant study‐wide. The risk ratios for morbidity, medication use, and health service visits were calculated using negative binomial regression models.

### 2.8 Ethical approval

The ethical committee of the Northern Ostrobothnia Hospital District approved the study (EETTMK 94/2011) on December 14, 2011. All the participants of NFBC1966 gave informed consent for the use of their collected data for scientific purposes.

## 3 RESULTS

### 3.1 Self‐reported diagnosis set by a doctor

After correction for multiple testing, the women with PCOS had a higher prevalence of hypertension, DMT2, depression, migraine, tendinitis, osteoarthritis in the knee, back or shoulder, fractures, gestational diabetes, pre‐eclampsia, and endometriosis compared with the non‐PCOS controls by age 46 years. After adjusting for the confounding factors (BMI, physical activity, alcohol consumption, smoking, marital status, and education), significance remained for hypertension, migraine, tendinitis, arthroses, fractures, and endometriosis. (Table 1, all diagnoses Supporting Information Table S1).

### 3.2 Overall morbidity and self‐reported health status

At age 46, the self‐reported health status was decreased in women with PCOS as 9.2% reported a poor or very poor health status compared with 3.1% of the controls (*P* < 0.001). In line with this, the women with PCOS presented with multimorbidity at age 46. The morbidity score distributions at age 46 in the PCOS cases and controls are presented in Figure 1B. The women with PCOS more often had five or more comorbidities, whereas controls more often had fewer than three (Figure 1B). Regarding different phenotypic groups, the women reporting diagnosis for PCOM/PCOS by the age of 46 years had higher median morbidity scores than controls, and the group reporting both oligo/amenorrhea + hirsutism at 31 years and PCOM/PCOS diagnosis at 46 years had the highest scores (Figure 1C). When considering the BMI of the study participants, we noted that both the normal weight (NW, BMI < 25 kg/m^2^) and the overweight/obese (OW/OB, BMI ≥25 kg/m^2^) women with PCOS had higher median morbidity scores than controls in the same weight group. Most importantly, the morbidity scores of NW vs OW/OB women with PCOS did not differ, whereas NW controls had lower morbidity scores than OW/OB controls (Figure 1D).

When comparing the morbidity scores calculated using the 68 questions asked in the questionnaires at age 46, women with PCOS had 35% higher morbidity risk (risk ratio [RR] 1.35, 95% confidence interval [CI] 1.16–1.57) (Figure 1E), which did not substantially change after adjusting for confounding factors (RR 1.37, 95% CI 1.17–1.60).

To validate if the morbidity score, specific for this study, truly reflected the health status of an individual, we conducted a correlation analysis with the self‐reported health status, a measure previously described to be associated with mortality and morbidity, and to predict survival better than medical records.^23^, ^24^, ^25^ The overall morbidity score correlated positively with self‐reported health (*r* = 0.338, *p* < 0.001), indicating that those with poorer perceived health also reported a higher number of morbidities.

### 3.3 Self‐reported symptoms regarding asthma, allergies, infections, and autoimmune diseases

At age 46, women with PCOS had more “cough with wheezing”, “recurrent respiratory infections”, and “atopic, infantile, or allergic eczema” than controls. PCOS cases also reported having “two or more pneumonias”, “recurrent otitis in adulthood”, “other health‐endangering recurrent infections”, and “common colds”, and being “more susceptible to infections than other people”. PCOS cases also reported more symptoms related to autoimmune diseases, such as “joint pain”, “joint swelling”, and “pain in the heel” than the controls. Adjustments did not affect the results (Table 2, all symptoms Supporting Information Table S1).

### 3.4 Self‐reported medications and risk for medication use

Regarding self‐reported medication use at the age of 46 years, the main medication groups that were more commonly used by women with PCOS than the controls were “alimentary tract and metabolism”, “dermatological”, “systemic hormonal preparations excluding sex hormones and insulins”, and “nervous system” after adjustment for confounding factors. As for the subgroup analysis, “drugs for functional gastrointestinal disorders”, “drugs used in diabetes”, “beta‐blocking agents”, “agents acting on the renin‐angiotensin system”, “antibiotics and chemotherapeutics for dermatological use”, “thyroid therapy”, and “analgesics” were more commonly used among women with PCOS compared with controls before and after adjustments, except for “beta‐blocking agents” (Table S1). Overall, women with PCOS had a 27% higher risk (RR 1.27, 95% CI 1.08–1.50) for medication use than controls (Figure 1D), which remained after adjustments (RR 1.21, 95% CI 1.01–1.46]).

### 3.5 Use of health services

Even though the median number of visits to any healthcare provider was higher in women with PCOS (median 7, interquartile range 3–12 vs median 5, interquartile range 3–10) than in controls (*p* = 0.010), adjustments for confounding factors rendered the risk nonsignificant (odds ratio 1.18, 95% CI 0.998–1.393).

## 4 DISCUSSION

This population‐based follow‐up study illustrates high multimorbidity risk and poor self‐rated health among women with PCOS until late reproductive years. We show PCOS associating with an increased risk for several diseases and symptoms, some of them linked, for the first time, to PCOS. Some of the differences in disease risk, and especially medication use, were driven by high BMI, indicating that PCOS, per se, may not always be the main cause for some of the comorbidities. Nevertheless, the median morbidity score of women with PCOS with a BMI of 25 kg/m^2^ or more was similar to that of women with PCOS and lower weight. More studies on pathomechanisms of the comorbidities in PCOS are warranted, as high BMI seems not to be solely responsible for the increased morbidity.

By the age of 46, the women with PCOS had a higher risk for DMT2, depression, migraine, hypertension, tendinitis, osteoarthroses (especially in knee, back, or shoulder), fractures, endometriosis, gestational diabetes, and pre‐eclampsia, although after adjustments the risks for DMT2, depression, gestational diabetes, and pre‐eclampsia were no longer significantly increased. Indeed, it has been established that normal‐weight women with PCOS are not at risk for developing DMT2, especially in Nordic populations.^6^ Metabolic conditions, such as hypertension and DMT2, are well established in PCOS, and they are also linked with the risk for pre‐eclampsia.^26^ A previous study has specifically assessed the cardiovascular diseases in this population, showing that hypertension risk is increased in PCOS, independent of obesity, and that CVD event risk increased until age 49 years.^8^ We have also reported adverse metabolic outcomes for women with PCOS, as well as registered cardiac morbidity.^5^, ^8^ Controversially, an increased long‐term CVD risk has not been detected in studies with populations of older age.^27^, ^28^

Our study also revealed an increased risk for migraine in PCOS, in line with the existing literature.^13^ Further mechanistic studies are warranted, as migraine is often related to menstrual cycle hormone fluctuations that are often disturbed in PCOS. Whether higher prevalence of endometriosis relates to more extensive gynecological assessments in this subfertile population, or prolonged estrogen action and progesterone resistance in PCOS, remains to be determined.^29^ Tendinitis has not been linked to PCOS before; however, musculoskeletal diseases in general and osteoarthritis are more common in the affected women.^14^, ^30^ The fracture risk among women with PCOS is debatable. In a Danish population, fractures were not more common,^13^ in contrast to a Taiwanese study,^31^ as well as ours.^32^ We have recently reported higher vitamin D levels in the same PCOS population;^33^ however, bone formation markers and bone mineral density seem to be decreased in PCOS.^32^, ^34^ Further studies should be carried out among women with PCOS with different phenotypes to segregate the role of hyperandrogenism and metabolic derangements.

Respiratory tract problems were more prevalent in women with PCOS. The women reported having cough with wheezing, recurrent respiratory infections, and atopic, infantile, or allergic eczema more often than the controls. The evidence of a higher prevalence of various respiratory infections and diseases in women with PCOS is increasing.^12^ The mechanisms behind these disorders are not known but increased systemic low‐grade inflammation^35^ or hyperandrogenism^36^ may be predisposing factors. The higher prevalence of eczemas is a novel finding, although some dermatological manifestations, such as hidradenitis suppurativa, have previously been linked to PCOS.^37^

This is the first study to assess self‐reported symptoms related to infections and autoimmune diseases among women with PCOS. The affected women more often reported recurrent infections, including pneumonias, otitis and common colds, and a higher susceptibility to infections than the controls at age 46 (Table 2). Also, symptoms related to autoimmune diseases were more common in women with PCOS than in controls (Table 2). These results are supported by a recent systematic review and meta‐analysis presenting women with PCOS having not only a higher risk of autoimmune thyroid disease^38^ but also a higher risk for asthma.^12^

Only one previous population‐based study on medication use among women with PCOS exists.^13^ Although the increased medication use in our study was self‐reported, the medication profile was similar to that reported in the Danish register‐based study. Medications used to treat alimentary tract and metabolic illnesses were more prevalent in PCOS. This group of medications include not only metformin, but also medications for gastric problems. The finding of increased use of drugs for functional gastrointestinal disorders may reflect higher stress and anxiety levels, which have been shown to result in gastrointestinal symptoms^39^ as well as increased risk for irritable bowel syndrome in PCOS.^40^ Use of medication targeting the cardiovascular system was higher in PCOS, as already reported in our previous publication.^8^ The use of beta blockers was higher in women with PCOS than in the controls. Given that the first‐line treatment for high blood pressure are medications targeting the renin–angiotensin system, it may be that the beta‐blocking agents were prescribed for indications other than hypertension,^41^ such as anxiety symptoms, which are highly prevalent in PCOS,^10^ as is sympathetic excitation.^42^ The higher use of medications affecting the “nervous system” in PCOS was expected, given the psychological morbidity related to the syndrome.^11^ Moreover, a higher use of medications to treat cutaneous manifestations was observed in PCOS, probably explained by acne, hirsutism, and male‐type hair loss, commonly related to the syndrome.^1^ As our study also showed an increased prevalence of atopic eczemas and other autoimmune symptoms in PCOS, more research should be targeted to dermatological manifestations related to the syndrome.

The women with PCOS reported morbidities, symptoms, and higher medication use more often than the controls. Moreover, women with PCOS estimated their health as poor or very poor almost three times more often compared with controls, in line with our previous finding.^20^ In line with this, the affected women reported more frequent healthcare visits, although this seemed mostly to be driven by high BMI. Even though an Australian study reported the hospitalization rate to be higher among women with PCOS,^14^ self‐reported health service visits have not been reported before. The finding that after adjustments the women with PCOS had not used more healthcare services within 12 months before the study, may be due to psychological factors, such as depression and anxiety, which may also impact the willingness of the women to seek help. It is worth noting that the selection criteria for the PCOS population represent most likely less severe PCOS phenotypes as women using hormonal contraceptives were excluded at age 31. Even though the women self‐reporting PCOS at age 46 included some of these women, there is still a chance of bias that underestimates the health‐burden related to PCOS.

The present study has many strengths, including the possibility to adjust for various confounding factors, which was possible because of the unique cohort data. The study screened symptoms for the first time, thus identifying conditions impossible to discern by International Classification of Diseases codes from the hospital registers. The self‐awareness bias was low because the birth cohort data collection did not target PCOS; rather, it focused on lifelong health and work ability. Indeed, the data represent participants from the same general community, with low variation in terms of ethnicity, education, or access to healthcare services, in comparison to selected PCOS populations from infertility clinics. Additionally, in Finland, there is very little variation in healthcare access, as the healthcare system is public and accessible to everyone regardless of area of residence. Although PCOS diagnosis might be considered a limitation, as mentioned above, the validity of the diagnosis has been well established in several previously published studies.^5^, ^10^, ^43^ Moreover, self‐reported symptoms have been shown to successfully identify women with PCOS.^43^ Moreover, the self‐report approach enables the identification of symptomatic women with PCOS who have not received the diagnosis, as the syndrome still remains underdiagnosed in health care.^17^ Despite statistical correction there may still be residue for multiple testing.

## 5 CONCLUSION

Our study emphasizes the multimorbidity and poor self‐rated health among women with PCOS. Further studies are warranted to explore the disease mechanisms in more detail.

## CONFLICT OF INTEREST

The authors have stated explicitly that there are no conflicts of interest in connection with this article.

## AUTHOR CONTRIBUTIONS

LK and TTP planned the study. LK, TTP, and RKA wrote the manuscript. LK, RKA, EK, and PP conducted the statistical analyses. RKA created the figures. SK, M‐RJ, SF, LM‐P, and JT provided help with the interpretation of the results. All the authors reviewed the manuscript.

## FUNDING

This study was supported by grants from The Finnish Medical Foundation, The Academy of Finland (315921, 321763), The Sigrid Juselius Foundation, The Finnish Cultural Foundation, The Jalmari and Rauha Ahokas Foundation, The Päivikki and Sakari Sohlberg Foundation, Genesis Research Trust (UK), The Medical Research Council, UK (G0802782), University of Oulu (65354, 24301140), Oulu University Hospital (2/97, 8/97, 24302240), Ministry of Health and Social Affairs (23/251/97, 160/97, 190/97), National Institute for Health and Welfare (54121), Regional Institute of Occupational Health, (50621, 54231), and ERDF European Regional Development Fund (539/2010 A31592).

## Supporting information


Figure S1

Table S1
Table S2.
Table S3.
Table S4.

## Data Availability

NFBC data are available from the University of Oulu, Infrastructure for Population Studies. Permission to use the data can be applied for research purposes via the electronic material request portal. In the use of data, we follow the EU general data protection regulation (679/2016) and Finnish Data Protection Act. The use of personal data is based on a cohort participant's written informed consent at his/her latest follow‐up study, which may cause limitations to its use. Please, contact NFBC project center (nfbcprojectcenter@oulu.fi) and visit the cohort website (www.oulu.fi/nfbc) for more information.
