# Supplementary figures and images for: Women with polycystic ovary syndrome are burdened with multimorbidity and medication use independent of body mass index at late fertile age: A population‐based cohort study
Source: Acta Obstet Gynecol Scand. 2022 Jun 8;101(7):728–36. doi: 10.1111/aogs.14382 (PMC9564432; doi:10.1111/aogs.14382)

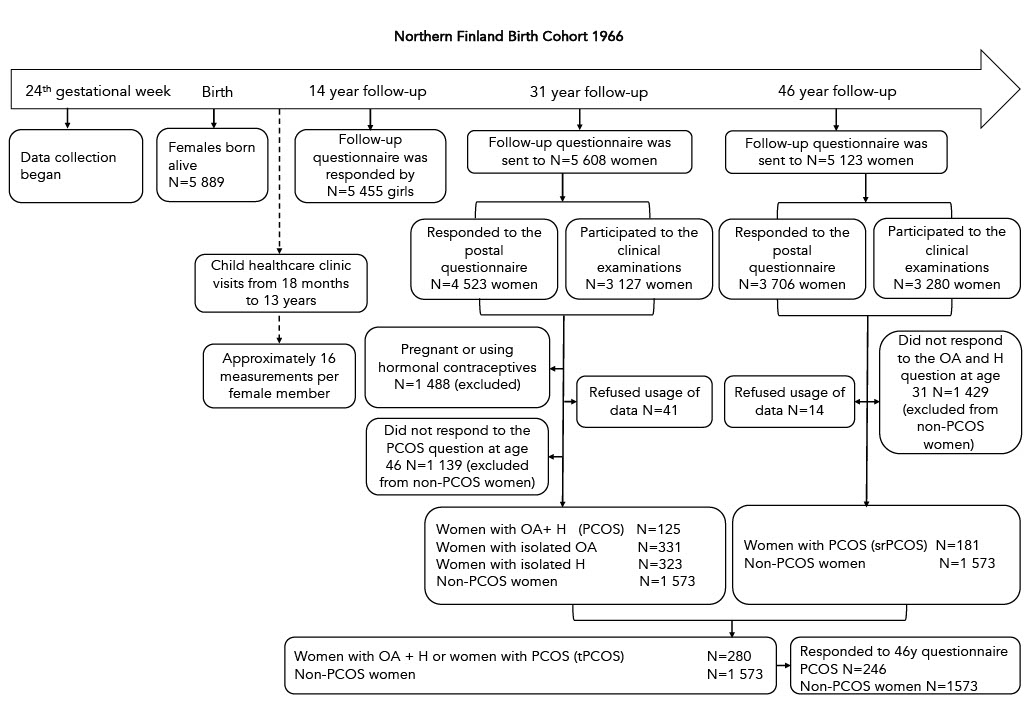

Supplement: Supplementary file 1 — Figure S1 [file AOGS-101-728-s001.jpg]
