# Supplementary material for: Women with polycystic ovary syndrome are burdened with multimorbidity and medication use independent of body mass index at late fertile age: A population‐based cohort study
Source: Acta Obstet Gynecol Scand. 2022 Jun 8;101(7):728–36. doi: 10.1111/aogs.14382 (PMC9564432; doi:10.1111/aogs.14382)
Supplement: Supplementary file 2 — Table S1 Table S2. Table S3. Table S4. [file AOGS-101-728-s002.docx]

**SUPPORTING INFORMATION**

**Table S1.** Medication use in women with PCOS and the controls at age 46 in the Northern Finland Birth Cohort 1966 (NFBC1966).

| **ATC MEDICATION GROUP** | | | Controls (1573) | PCOS  (241) | | cOR  (CI 95%)^a^ | | aOR  (CI 95%)^a^ |
| --- | --- | --- | --- | --- | --- | --- | --- | --- |
| **A: ALIMENTARY TRACT AND METABOLISM** | | | 338 (21.5%) | 73 (26.1%) | | 1.29 (0.96-1.73) | | **1.49 (1.10-2.04)*** |
| A3: Drugs for functional gastrointestinal disorders | | | 14 (0.9%) | 7 (2.9%) | | **3.33 (1.33-8.34)*** | | **3.45 (1.35-8.83)*** |
| A10: Drugs used in diabetes | | | 16 (1.0%) | 15 (6.2%) | | **6.46 (3.15-13.24)*** | | **4.54 (2.11-9.79)*** |
| **C: CARDIOVASCULAR SYSTEM** | | | 260 (16.5%) | 61 (21.8%) | | **1.41 (1.03-1.92)** | | 1.40 (0.99-1.98) |
| C7: Beta blocking agents | | | 104 (6.6%) | 29 (12.0%) | | **1.93 (1.25-2.99)*** | | **1.63 (1.04-2.58)** |
| C9: Agents acting on the renin-angiotensin system | | | 119 (7.6%) | 34 (14.1%) | | **2.01 (1.34-3.02)*** | | **1.70 (1.10-2.63)*** |
| **D: DERMATOLOGICALS** | | | 66 (4.2%) | 20 (7.1%) | | **1.76 (1.05-2.95)** | | **2.13 (1.26-3.61)*** |
| D6: Antibiotics and chemotherapeutics for dermatological use | | | 4 (0.3%) | 4 (1.7%) | | **6.62 (1.65-26.65)*** | | **6.67 (1.61-27.58)*** |
| D7: Corticosteroids, dermatological preparations | | | 55 (3.5%) | 15 (6.2%) | | **1.83 (1.02-3.30)** | | **1.90 (1.05-3.46)** |
| **H: SYSTEMIC HORMONAL PREPARATIONS EXCLUDING SEX HORMONES AND INSULINS** | | | 120 (7.6%) | 30 (10.7%) | | 1.47 (0.96-2.24) | | **1.67 (1.08-2.59)*** |
| H3: Thyroid therapy | | | 100 (6.4%) | 27 (11.2%) | | **1.86 (1.19-2.91)*** | | **1.77 (1.11-2.81)*** |
| **N: NERVOUS SYSTEM** | | | 503 (32.0%) | 109 (38.9%) | | **1.35 (1.04-1.76)** | | **1.69 (1.27-2.24)*** |
| N2: Analgesics | | | 326 (20.7%) | 72 (29.9%) | | **1.63 (1.21-2.20)*** | | **1.65 (1.21-2.24)*** |
| N6: Psychoanaleptics | | | 150 (9.5%) | 34 (14.1%) | | **1.56 (1.05-2.32)** | | 1.48 (0.98-2.25) |
|  |  |  | | |  | |  |  |

The results are reported as crude odds ratios (cORs) and adjusted odds ratios (aORs) with 95% confidence intervals (CIs), with and without adjustments (BMI, physical activity, alcohol consumption, smoking, marital status and education). The bolded values indicate significance (p<0.05) and * even after Benjamini-Hochberg correction.

**Table S2.** Population characteristics.

|  | Controls  (n=1308-1569) | PCOS  (n=205-280) | p-value |
| --- | --- | --- | --- |
| **BMI (kg/m^2^)** | 25.26[22.64;28.99] | 27.20[23.99;31.44] | **<0.001^a^** |
| **BMI, (kg/m^2^)**  normal weight (<25)n(%)  overweight (25-30) n(%)  obese (>30) n(%) | 775 (50.2%)  470 (30.4%)  299 (19.4%) | 92 (38.7%)  74 (31.1%)  72 (30.3%) | **<0.001^b^** |
| class I (30-34.99)n(%all OB) | 209 (69.9%) | 40 (55.6%) | 0.054^b^ |
| class II (35-39.99) n(%all OB) | 72 (24.1%) | 24 (33.3%) |  |
| class III (≥40) n(%all OB) | 18 (6.0%) | 8 (11.1%) |  |
| **Physical activity** | 13.33[7.06;25.00] | 13.75[6.00;25.63] | 0.801^a^ |
| **Alcohol consumption**  abstinence n(%)  low-risk drinking n(%)  high-risk drinking n(%) | 192 (12.3%)  1248 (79.8%)  124 (7.9%) | 37 (15.4%)  184 (76.3%)  20 (8.3%) | 0.397^b^ |
| **Smoking**  no smoking n(%)  previous/occasional smoking n(%)  regular smoking n(%) | 886 (56.9%)  346 (22.2%)  325 (20.9%) | 130 (55.1%)  49 (20.8%)  57 (24.2%) | 0.524^b^ |
| **Marital status**  in a relationship n(%)  single n(%) | 1215 (77.7%)  351 (22.3%) | 234 (83.6%)  46 (16.4%) | **0.027^b^** |
| **Education**  basic  secondary  tertiary | 86 (5.5%)  998 (63.5%)  489 (31.1%) | 19 (7.9%)  157 (65.1%)  65 (27.0%) | 0.189^b^ |

Clinical features in women with polycystic ovary syndrome (PCOS) and the controls at age 46. Data presented as medians [quartiles] and percentages. The number of women varied between the analyses; some of the women who completed the questionnaire did not attend the clinical examination.

^a^Mann-Whitney U-test, ^b^Pearson’s Chi-square test.

**Table S3.** All self-reported diagnoses at age 46.

|  | **46 years** | | | |  |
| --- | --- | --- | --- | --- | --- |
| **Self-reported diagnosis** | **Control N (%)** | **PCOS N (%)** | **cOR (95 % CI)** | **aOR (95% CI)** |  |
|  |  |  |  |  |  |
| Chlamydia infection | 158 (10.1%) | 24 (10.0%) | 0.98 (0.63-1.55) | 1.05 (0.66-1.66) |  |
| Herpesviral infection of genitalia | 77 (4.9%) | 14 (5.8%) | 1.20 (0.67-2.15) | 1.41 (0.77-2.56) |  |
| Condyloma | 224 (14.3%) | 39 (16.3%) | 1.17 (0.80-1.69) | 1.25 (0.85-1.84) |  |
| Other genital infection | 109 (7.0%) | 19 (7.9%) | 1.15 (0.69-1.90) | 1.22 (0.73-2.05) |  |
| Leiomyoma of uterus | 300 (19.2%) | 51 (21.5%) | 1.16 (0.83-1.62) | 1.20 (0.85-1.68) |  |
| Cancer | 47 (3.0%) | 8 (3.4%) | 1.15 (0.53-2.46) | 1.15 (0.53-2.50) |  |
| Anaemia | 284 (18.4%) | 54 (22.8%) | 1.31 (0.94-1.82) | 1.37 (0.98-1.93) |  |
| Hypothyroidism | 109 (7.0%) | 28 (11.7%) | **1.76 (1.13-2.73)** | **1.75 (1.12-2.74)** |  |
| Hyperthyroidism | 22 (1.4%) | 8 (3.4%) | **2.53 (1.11-5.77)** | **2.46 (1.02-5.97)** |  |
| Diabetes type 1 | 3 (0.2%) | 1 (0.4%) | 2.18 (0.23-21.06) | 1.81 (0.17-18.83) |  |
| Diabetes type 2 | 34 (2,2%) | 16 (6.7%) | **3.21 (1.74-5.91)*** | **2.40 (1.25-4.63)** |  |
| Psychosis | 19 (1.2%) | 6 (2.5%) | 2.22 (0.87-5.64) | 2.38 (0.90-6.27) |  |
| Depression | 219 (14.0%) | 49 (20.5%) | **1.58 (1.12-2.23)*** | **1.57 (1.09-2.25)** |  |
| Other mental health problem | 78 (5.0%) | 11 (4.6%) | 0.93 (0.49-1.78) | 0.86 (0.44-1.70) |  |
| Epilepsy | 23 (1.5%) | 5 (2.1%) | 1.36 (0.51-3.59) | 1.16 (0.43-3.09) |  |
| Migraine | 386 (24.7%) | 82 (34.2%) | **1.59 (1.19-2.12)*** | **1.58 (1.17-2.13)*** |  |
| Sleep apnoea | 19 (1.2%) | 3 (1.3%) | 1.02 (0.30-3.47) | 0.81 (0.23-2.82) |  |
| Other neurological condition | 26 (1.7%) | 32 (13.4%) | **2.57 (1.22-5.40)** | **2.79 (1.31-5.96)** |  |
| Hypertension | 230 (17.9%) | 73 (30.4%) | **2.00 (1.48-2.71)*** | **1.76 (1.27-2.44)*** |  |
| Angina pectoris | 8 (0.5%) | 3 (1.3%) | 2.81 (0.72-10.95) | 2.64 (0.66-10.51) |  |
| Cardiac insuffiency | 6 (0.4%) | 2 (0.8%) | 1.86 (0.38-8.98) | 1.71 (0.34-8.54) |  |
| Stroke | 16 (1.0%) | 4 (1.7%) | 1.63 (0.54-4.93) | 1.60 (0.52-4.91) |  |
| Gastric/duodenal ulcer | 33 (2.1%) | 11 (4.6%) | **2.23 (1.11-4.47)** | **2.12 (1.01-4.45)** |  |
| Hernia | 80 (5.2%) | 15 (6.3%) | 1.24 (0.70-2.19) | 1.10 (0.61-1.99) |  |
| Crohn disease/ulcerative colitis | 42 (2.7%) | 7 (3.0%) | 1.10 (0.49-2.47) | 1.19 (0.52-2.72) |  |
| Gallstones, gallbladder infections | 105 (6.7%) | 32 (13.4%) | **1.70 (1.08-2.67)** | 1.48 (0.92-2.37) |  |
| Coeliac disease | 41 (2.6%) | 3 (1.3%) | 0.48 (0.15-1.57) | 0.49 (0.15-1.62) |  |
| Hand dermatitis | 260 (16.6%) | 43 (18.1%) | 1.12 (0.78-1.60) | 1.07 (0.74-1.55) |  |
| Psoriasis | 37 (2.4%) | 5 (2.1%) | 0.87 (0.34-2.24) | 0.85 (0.32-2.22) |  |
| Arthropathic psoriasis | 8 (0.5%) | 1 (0.4%) | 0.82 (0.10-6.57) | 0.85 (0.10-7.11) |  |
| Other skin disease | 182 (11.8%) | 35 (14.9%) | 1.31 (0.89-1.94) | 1.34 (0.90-2.00) |  |
| Reactive arthropathy | 13 (0.8%) | 2 (0.8%) | 1.08 (0.24-4.88) | 1.10 (0.24-5.02) |  |
| Rheumatoid arthritis | 24 (1.5%) | 5 (2.1%) | 1.36 (0.52-3.61) | 1.51 (0.56-4.07) |  |
| Gout | 14 (0.9%) | 2 (0.8%) | 0.93 (0.21-4.13) | 0.93 (0.20-4.19) |  |
| Fibromyalgia | 35 (2.2%) | 12 (5.0%) | **2.30 (1.18-4.49)** | **2.20 (1.09-4.45)** |  |
| Ankylosing spondylitis | 6 (0.4%) | 3 (1.2%) | 2.81 (0.72-10.94) | 3.06 (0.77-12.24) |  |
| Osteoporosis | 16 (1.0%) | 3 (1.2%) | 1.30 (0.38-4.54) | 1.47 (0.41-5.20) |  |
| Tendinitis | 150 (9.6%) | 40 (16.9%) | **1.91 (1.30-2.95)*** | **1.81 (1.22-2.68)*** |  |
| Degenerative or other back condition | 288 (18.6%) | 55 (23.0%) | 1.31 (0.94-1.82) | 1.30 (0.93-1.82) |  |
| Arthrosis of hip | 29 (1.9%) | 7 (2.9%) | 1.52 (0.66-3.50) | 1.58 (0.67-3.74) |  |
| Arthrosis of back | 52 (3.4%) | 17 (7.1%) | **2.15 (1.22-3.78)*** | **2.34 (1.31-4.18)*** |  |
| Arthrosis of knee | 94 (6.1%) | 31 (13.0%) | **2.29 (1.49-3.53)*** | **2.26 (1.45-3.52)*** |  |
| Arthrosis of finger | 72 (4.7%) | 11 (4.6%) | 0.98 (0.51-1.88) | 0.96 (0.49-1.85) |  |
| Arthrosis of temporomandibular joint | 19 (1.2%) | 2 (0.8%) | 0.71 (0.17-3.10) | 0.56 (0.13-2.49) |  |
| Arthrosis of ankle joint | 17 (1.1%) | 4 (1.7%) | 1.53 (0.51-4.58) | 1.82 (0.58-5.76) |  |
| Arthrosis of foot | 20 (1.3%) | 3 (1.3%) | 0.97 (0.29-3.29) | 0.86 (0.25-2.94) |  |
| Arthrosis of shoulder | 34 (2.2%) | 13 (5.5%) | **2.58 (1.34-4.97)*** | **2.71 (1.38-5.30)*** |  |
| Other arthrosis | 50 (3.4%) | 9 (4.0%) | 1.18 (0.57-2.44) | 1.24 (0.60-2.60) |  |
| Other rheumatoid condition | 38 (2.4%) | 6 (2.5%) | 1.13 (0.43-2.95) | 1.19 (0.45-3.14) |  |
| Other arthritis condition | 29 (2.0%) | 5 (2.2%) | 1.81 (0.97-3.40) | 1.12 (0.46-2.73) |  |
| Fractures | 263 (16.8%) | 58 (24.5%) | **1.61 (1.16-2.22)*** | **1.73 (1.24-2.41)*** |  |
| Pyelonephritis^ | 113 (7.2%) | 24 (10.0%) | 1.42 (0.90-2.26) | 1.44 (0.90-2.30) |  |
| Salpingo-oophoritis | 47 (3.0%) | 15 (6.3%) | **2.16 (1.19-3.93)** | **2.14 (1.16-3.97)** |  |
| Endometriosis | 131 (8.4%) | 32 (13.4%) | **1.70 (1.12-2.56)** | **1.81 (1.19-2.76)*** |  |
| Gestational diabetes | 344 (25.1%) | 65 (30.2%) | **2.00 (1.48-2.71)*** | 1.52 (0.97-2.37) |  |
| Pre-eclampsia | 117 (8.7%) | 33 (15.5%) | **1.94 (1.28-2.94)*** | **1.76 (1.15-2.71)** |  |
| Congenital heart disease | 38 (2.4%) | 5 (2.1%) | 0.88 (0.344-2.27) | 1.02 (0.39-2.66) |  |
| Injury of tendon | 69 (4.4%) | 14 (5.9%) | 1.37 (0.76-2.48) | 1.37 (0.75-2.51) |  |
| Problems with alcohol | 19 (1.2%) | 4 (1.7%) | 1.38 (0.47-4.09) | 1.23 (0.39-3.83) |  |
| Other intoxicant problem | 6 (0.4%) | 2 (0.8%) | 2.18 (0.44-10.86) | 1.87 (0.36-9.62) |  |
| Other sickness/injury | 289 (19.7%) | 51 (23.1%) | 1.22 (0.87-1.71) | 1.32 (0.93-1.86) |  |

The results are reported as crude odds ratios (cORs) with 95% confidence intervals (CIs) and adjusted odds ratios (aORs) with 95% confidence intervals (CIs). The ORs in bold were statistically significant and with * also after Benjamini-Hochberg correction. Confounding factors used for adjustments: BMI, physical activity, alcohol consumption, smoking, marital status and education.

**Table S4.** All self-reported symptoms concerning asthma, allergies, infections and autoimmune symptoms at age 46.

|  | **46 years** | | |  |
| --- | --- | --- | --- | --- |
|  | **Controls** | **PCOS** | **cOR (95% CI)** | **aOR (95% CI)** |
| **Questions concerning asthma and allergy** |  |  |  |  |
| Asthma | 233 (15.4%) | 48 (21.1%) | **1.47 (1.04-2.08)** | **1.47 (1.03-2.09)** |
| Cough with wheezing | 450 (29.5%) | 98 (41.9%) | **1.72 (1.30-2.28)*** | **1.70 (1.27-2.28)*** |
| Recurrent respiratory infections | 429 (28.6%) | 85 (37.9%) | **1.53 (1.14-2.05)*** | **1.54 (1.15-2.08)*** |
| Emphysema, chronic bronchitis | 68 (4.5%) | 16 (6.9%) | 1.58 (0.90-2.77) | 1.62 (0.92-2.87) |
| Allergic rhinitis | 655 (42.9%) | 112 (48.3%) | 1.24 (0.94-1.64) | 1.21 (0.91-1.60) |
| Atopic, infantile or allergic eczema | 433 (28.3%) | 84 (36.4%) | **1.45 (1.08-1.93)*** | **1.45 (1.08-1.95)*** |
| Allergic eye symptoms | 631 (41.2%) | 108 (47.4%) | 1.29 (0.97-1.70) | 1.26 (0.95-1.67) |
|  |  |  |  |  |
| **Questions concerning infection symptoms** |  |  |  |  |
| Pneumonia at least twice | 137 (8.8%) | 37 (15.4%) | **1.88 (1.27-2.78)*** | **1.91 (1.28-2.86)*** |
| Hospitalization due to recurrent infections | 180 (11.5%) | 38 (15.8%) | 1.44 (0.99-2.11) | **1.49 (1.02-2.19)** |
| Sinus operation due to recurrent infections | 64 (4.1%) | 13 (5.4%) | 1.32 (0.72-2.43) | 1.36 (0.73-2.52) |
| Recurrent otitis in adulthood | 67 (4.3%) | 25 (10.4%) | **2.55 (1.58-4.12)*** | **2.53 (1.53-4.17)*** |
| Other health endangering recurrent infections | 31 (2.0%) | 11 (4.6%) | **2.29 (1.14-4.61)*** | **2.27 (1.11-4.63)*** |
| More common colds | 97 (6.2%) | 25 (10.4%) | **1.75 (1.10-2.78)*** | **1.67 (1.28-3.54)*** |
| More susceptible to infections than other people | 74 (4.7%) | 22 (9.2%) | **2.07 (1.26-3.40)*** | **2.13 (1.28-3.54)*** |
| Reduced defense capability against infections in relatives | 30 (1.9%) | 6 (2.5%) | 1.32 (0.54-3.20) | 1.29 (0.53-3.17) |
|  |  |  |  |  |
| **Questions concerning autoimmune symptoms** |  |  |  |  |
| Dry eyes | 599 (38.2%) | 103 (42.7%) | 1.21 (0.92-1.59) | 1.25 (0.95-1.65) |
| Dry mouth | 208 (13.3%) | 35 (14.6%) | 1.11 (0.75-1.63) | 1.09 (0.74-1.61) |
| Raynaud's phenomenon | 88 (5.6%) | 18 (7.5%) | 1.37 (0.81-2.31) | 1.30 (0.77-2.22) |
| Solar dermatitis | 469 (29.9%) | 69 (28.6%) | 0.94 (0.70-1.27) | 0.98 (0.72-1.33) |
| Skin burning easily in the sun | 529 (33.7%) | 85 (35.6%) | 1.09 (0.82-1.44) | 1.11 (0.83-1.48) |
| Mild fever, over 37 degrees | 121 (7.7%) | 28 (11.6%) | **1.57 (1.02-2.43)** | **1.74 (1.11-2.71)** |
| Low white cell count | 42 (2.7%) | 9 (3.8%) | 1.40 (0.68-2.92) | 1.47 (0.70-3.07) |
| Thrombosytopenia | 44 (2.8%) | 9 (3.8%) | 1.37 (0.66-2.85) | 1.37 (0.66-2.87) |
| Joint pain | 572 (36.4%) | 113 (47.3%) | **1.57 (1.19-2.06)*** | **1.46 (1.10-1.94)*** |
| Joint swelling | 240 (15.3%) | 53 (22.2%) | **1.57 (1.13-2.20)*** | **1.49 (1.05-2.11)*** |
| Pain of the heel | 278 (17.8%) | 71 (29.6%) | **1.94 (1.43-2.64)*** | **1.75 (1.26-2.41)*** |
| Sausage-shaped swelling of the fingers/toes | 128 (8.2%) | 29 (12.1%) | **1.54 (1.00-2.36)** | 1.34 (0.85-2.11) |

The results are reported as crude odds ratios (cORs) and adjusted (BMI, physical activity, alcohol consumption, smoking, marital status and education) odds ratios (aORs) with 95% confidence intervals (CIs). The values in bold and with * were statistically significant after adjustments and Benjamini-Hochberg correction.
